# A Comparative Study of Topical Procapil With Platelet-Rich Plasma Therapy Versus Topical Redensyl, Saw Palmetto, and Biotin With Platelet-Rich Plasma Therapy in the Treatment of Androgenetic Alopecia

**DOI:** 10.7759/cureus.38696

**Published:** 2023-05-08

**Authors:** Pavithra TR, Rajashekar TS, Suresh Kumar K, Harish Prasanna

**Affiliations:** 1 Dermatology, Venereology, and Leprosy, Sri Devaraj Urs Academy of Higher Education and Research, Kolar, IND; 2 Dermatology, Sri Devaraj Urs Academy of Higher Education and Research, Kolar, IND

**Keywords:** saw palmetto, combination therapy, procapil, platelet-rich plasma (prp), male androgenetic alopecia

## Abstract

Background and aim

Androgenetic alopecia (AGA) is a well-known hair loss disorder in both men and women affecting approximately 80% and 50% of the population, respectively. Various treatment options for AGA are available with varying efficacy. Combination therapy is a new dictum to combat AGA. Hence, this study aimed to compare the efficacy of commonly used topical therapies such as Procapil with platelet-rich plasma (PRP) and redensyl, saw palmetto (SP), and biotin (RSB) with PRP.

Materials and methods

This was a randomized controlled trial conducted on 54 male patients with AGA attending the outpatient department in a tertiary care hospital. Participants were randomly assigned into two equal groups (A and B). Group A participants were treated with Procapil with PRP, and group B participants were treated with redensyl, saw palmetto, and biotin with PRP at three weeks intervals for a total period of four sessions. Clinical improvement was assessed by serial hair photography by a third blinded observer and was recorded.

Results

A total of 54 individuals were included and were distributed into 27 each in group A and group B. AGA grading score was found significant between the groups with P* *< 0.05.

Conclusion

PRP with adjuvants redensyl, saw palmetto, and biotin can be a better alternative to the current therapies of PRP.

## Introduction

Androgenic alopecia (AGA) is a common progressive disorder that leads to hair loss, which affects men more often than women. The polygenic status of AGA varies in severity according to the age of onset and the location of hair loss on the scalp. Moreover, the incidence of AGA is approximately 30% by age 30, 50% by age 50, and 80% by age 70. AGA is characterized by the progressive miniaturization of the hair follicle, resulting in the transformation of terminal hairs into vellus hairs [1].

AGA is an androgen-dependent condition in which testosterone is converted to dihydrotestosterone (DHT) by 5α-reductase. Dihydrotestosterone binds to the androgen receptors and triggers genes that convert healthy terminal hair into vellus hair, primarily in androgen-dependent areas of the scalp (i.e., frontal, parietal, and vertex) [2].

Treatment options for AGA depend on many factors, including efficacy, feasibility, risks, and costs. Additionally, AGA patients seek treatment for cosmetic reasons. It is a common problem, especially in men, and has been treated using a variety of topical, oral, surgical, and implant therapies [3]. However, the US Food and Drug Administration (FDA) has approved only minoxidil and oral finasteride for the treatment of AGA [3]. Although these drugs may effectively control AGA, their disadvantages include being expensive, requiring lifelong treatment, and having side effects such as vasodilation and 5α-reductase inhibition [4].

On the other hand, redensyl is prepared from a combination of botanical ingredients and contains dihydroquercetin glucoside (DHQG) (0.005%), epigallocatechin gallate glucoside (EGCG2) (0.0009%), glycine (0.005%), zinc chloride (0.002%), metabisulfite (0.015%), and glycerin (50%), among which DHQG and EGCG2 (two stabilized polyphenols) target and stimulate fibroblasts in the outer root sheath stem cells and dermal papilla, whereas glycine and zinc are required for hair metabolism [5].

Biotin (vitamin B7 or H) is converted to biotinyl-GHK, a vitamin-carrying peptide. Deficiency of this causes baldness, sagging of skin, dermatitis, and hair loss [6].

Moreover, Procapil contains three highly effective botanical ingredients: oleanolic acid, which is extracted from olive leaves and inhibits the enzymes 5-1 and 5-2 alpha-reductase; apigenin, a flavonoid extracted from citrus fruit peels, which helps dilate blood vessels; and the peptide glycine-histidine-lysine, which is required for pro-matrix metalloproteinase activity and is important in providing the metabolic needs of the hair [7].

Additionally, the fruit of *Serenoa repens*, a dwarf tree native to the subtropics of the southern United States, is used to produce the saw palmetto (SP) plant extract, which is a competitive, nonselective inhibitor of 5α-reductase isoforms that both blocks dihydrotestosterone (DHT) nuclear uptake and reduces DHT-binding capacity at the androgen receptor by nearly 50%. As such, it is widely used as an adjunctive treatment for alopecia due to its antiandrogenic properties, low risk of side effects, and low potential for drug interactions [8].

From the data presented, it can be deduced that alternative medicine for AGA is being researched, and treatment options such as Procapil and RSB are being utilized in practice. In addition, platelet-rich plasma (PRP) therapy is one of the treatment options for AGA [5]. However, despite positive practical experiences with the use of PRP, the combination therapy of PRP with Procapil and “redensyl, saw palmetto, and biotin (RSB)” is lacking, which means that the treatment options for AGA are still inadequate for good evidence-based treatment.

This study was proposed to assess and compare the clinical efficacy and safety of PRP with topical Procapil and a topical combination of RSB for AGA.

## Materials and methods

Methodology

This randomized controlled study was conducted at the Department of Dermatology, Venereology, and Leprosy for a period of six months from August 2022 to February 2023. The study was approved by the ethical committee of the hospital (ethical committee approval number: DMC/KLR/IEC/187/2022-23). Patients with androgenetic alopecia in the male population were enrolled in the study after obtaining informed consent. Patients with endocrine disorders, metabolic disorders, platelet disorders, hypertension, and cardiovascular problems were excluded. Patients were counseled with respect to the side effects and limitations of undertaking the treatment.

A total of 54 AGA patients were randomized into two equal groups, group A and group B (27 individuals in each group), using a computer-generated block randomization from www.randomization.com [9]. Group A included patients treated with topical Procapil with PRP therapy, and group B included patients treated with topical RSB with PRP therapy. Patients in both groups were treated with four sessions of appropriate therapy at three-week intervals and were followed up at the end of the third and sixth weeks.

Group A patients received PRP once every three weeks and applied 1 mL of topical Procapil daily. Patients in group B received PRP once every three weeks and applied 1 mL topical combination of redensyl, saw palmetto, and biotin once daily.

Participants were required to participate in serial global photography at baseline and at subsequent PRP sessions. A third observer assessed the treatment efficacy based on the global photographic assessment (GPA) scale during which lighting and position were identical. Scores of 0, 1, 2, and 3 were assigned by the third observer for <25%, 26%-50%, 51%-75%, and >75% improvement, respectively. Clinical assessment of AGA was performed using the Norwood-Hamilton classification for men (grades I to VII) at baseline and at subsequent visits for PRP treatment every three weeks for all four sessions. Follow-up was done at the end of the third and sixth week after the completion of the therapy, and side effects were noted. A visual analog scale (VAS) (scores from 0 to 10) was used to assess the participants’ perceived satisfaction with the treatment at the end of the second follow-up.

Statistical analysis

Data were analyzed using Microsoft Excel (Microsoft Corp., Redmond, WA, USA), and statistics were calculated using the Statistical Package for the Social Sciences (SPSS) (IBM SPSS Statistics, Armonk, NY, USA). Categorical data were represented in the form of frequencies and proportions. Moreover, chi-square was used as a test of significance. Continuous data were represented as mean and standard deviation. A P-value < 0.05 was considered statistically significant.

## Results

As shown in Table 1, of the total of 54 patients included in the study, most of the patients belonged to the age group of 21-30 years (53.7%), followed by 31-40 years (35.2%). Among the 54 patients, 55.6% (n = 30) had a positive family history of AGA, whereas 44.4% (n = 24) had no significant family history.

**Table 1:** Distribution of patients according to age

Age group (years)	Number of patients (%)		Total (N = 54) (%)
	Group A (n = 27)	Group B (n = 27)	
21-30 years	17 (63%)	12 (44.4%)	29 (53.7%)
31-40 years	6 (22.2%)	13 (48.1%)	19 (35.2%)
41-50 years	4 (14.8%)	2 (7.4%)	6 (11.1%)

As depicted in Table 2, most of the patients belonged to grade III of Norwood-Hamilton AGA grading, which accounts for 44.4% (n = 24), followed by grades II, IV, and I, accounting for 33.3% (n = 18), 20.4% (n = 11), and 1.8% (n = 1) in our study, respectively.

**Table 2:** AGA grading according to the Norwood-Hamilton classification AGA: androgenetic alopecia

Norwood-Hamilton grade at baseline	Group A (n = 27) (%)	Group B (n = 27) (%)	Total (N = 54) (%)
Grade I	1 (3.7%)	0 (0%)	1 (1.8%)
Grade II	12 (44.4%)	6 (22.2%)	18 (33.3%)
Grade III	8 (29.6%)	16 (59.3%)	24 (44.4%)
Grade IV	6 (22.2%)	5 (18.5%)	11 (20.4%)

In group A, 18.5% (n = 5) of the patients had a significant reduction in AGA grading based on the Norwood-Hamilton classification, whereas 25.9% (n = 7) of patients in group B showed a significant reduction in AGA grading based on the Norwood-Hamilton classification.

As described in Table 3, the mean AGA improvement percentage was 11.9% in group A and 21.9% in group B. These values are statistically significant (P-value < 0.00001).

**Table 3:** Mean AGA improvement based on the Norwood-Hamilton classification AGA: androgenetic alopecia, BL: baseline presentation, 1: at the end of the first session, 2: at the end of the second session, 3: at the end of the third session, 4: at the end of the fourth session, FU1: at the end of the first follow-up session, FU2: at the end of the second follow-up session

	BL	1	2	3	4	FU1	FU2	Mean AGA improvement (%)
Group A	2.9	2.7	2.44	2.7	2.44	2.37	2.51	11.9%
Group B	2.74	2.81	2.85	2.96	2.33	2.77	2.14	21.9%
P-value								<0.00001

In the efficacy of both treatment modalities assessed using the GPA scale at the end of the second follow-up, 7.4% (n = 2) and 22.2% (n = 6) showed a score of 3 (>75% improvement) in group A and group B, respectively.

Table 4 shows the results of the GPA scale where the values are statistically significant.

**Table 4:** Efficacy assessed using the GPA scale GPA: global photographic assessment, 1: at the end of the first session, 2: at the end of the second session, 3: at the end of the third session, 4: at the end of the fourth session, FU1: at the end of the first follow-up session, FU2: at the end of the second follow-up session

	1	2	3	4	FU1	FU2
Group A	0	0.44	0.81	1.22	1.44	1.48
Group B	0	0.51	1	1.42	1.73	2.03
P-value		<0.00001	<0.00001	<0.00001	<0.00001	<0.00001

Patient satisfaction was assessed in both treatment modalities using a visual analog scale at the end of the second follow-up; 25.9% (n = 7) in group A and 44.4% (n = 12) in group B showed a VAS score of more than or equal to (≥) 8. The mean VAS of group A and group B were 6.8 and 7.4, respectively. These results are statistically significant.

As shown in Table 5, the most notable side effect was pruritus found in 14.8% (n = 8) of the participants, followed by allergic contact dermatitis and seborrheic dermatitis accounting for 13% (n = 7) and 9.2% (n = 5), respectively. Most of the patients had no side effects.

**Table 5:** Side effects

	No side effects	Pruritus	Seborrheic dermatitis	Allergic contact dermatitis
Group A	16	5	2	4
Group B	18	3	3	3
Total	34 (63%)	8 (14.8%)	5 (9.2%)	7 (13%)

**Figure 1:**
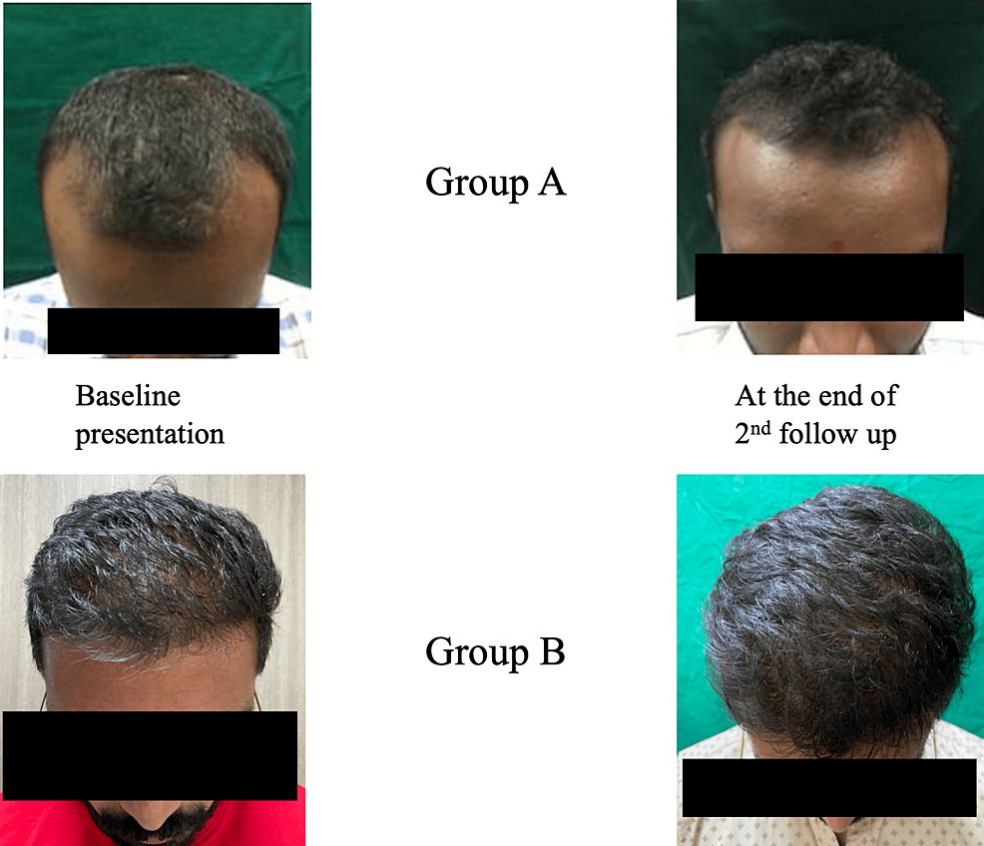
Efficacy of topical Procapil with platelet-rich plasma therapy (group A) versus topical redensyl, saw palmetto, and biotin with platelet-rich plasma therapy (group B) in the treatment of androgenetic alopecia

Figure 1 shows the efficacy of topical Procapil with platelet-rich plasma therapy versus topical redensyl, saw palmetto, and biotin with platelet-rich plasma therapy in the treatment of androgenetic alopecia.

## Discussion

AGA has a progressive course and is associated with significant side effects that adversely affect the quality of life (QOL), body image, self-esteem, and emotional health and may trigger neurotic conditions such as anxiety and depression [8]. It is, therefore, important to provide patients with this disorder with the best possible care.

Although there are several scientifically proven treatments for AGA, including topical agents, oral agents, and procedures (PRP, hair transplants, etc.), only a few studies are available regarding the combination therapy of PRP with RSB and Procapil.

PRP is a cocktail of growth factors in higher concentration, which is a product of autologous blood that improves the survival of dermal papilla cells during the hair cycle and also upregulates the fibroblast growth factor-7 [10]. Procapil inhibits the 5-1 and 5-2 alpha-reductase enzymes [7]. Redensyl targets and stimulates fibroblasts in the outer root sheath stem cells and the dermal papilla [5]. Biotin (also known as vitamin B7 or vitamin H) is a water-soluble vitamin that serves as an essential cofactor for carboxylase enzymes in multiple metabolic pathways [11]. SP is a competitive, nonselective inhibitor of 5α-reductase isoforms that both blocks dihydrotestosterone (DHT) nuclear uptake and reduces DHT-binding capacity at the androgen receptor by nearly 50%. Hence, in the present study, the combinations of PRP with other adjuvants were attempted.

Most of the participants in the present study were between the ages of 21 and 30 (52%), followed by those between the ages of 31 and 40 (35%). This result was consistent with the study by Butt et al. [12], in which a high proportion of the age group was between 21 and 25 years of age. This might be because most of them in this age group are more concerned about their aesthetic appeal. Accounting for 55.6% (n = 30), the majority had a positive family history of AGA, which is comparable to the studies by Butt et al. [12] and Pachar et al. [13].

The most morphologic type in our study was Norwood-Hamilton grade III (44%), followed by grades II and IV (33% and 20.4%, respectively). This was comparable to other studies by Butt et al. [12] and Rossi et al. [14], recording Norwood-Hamilton grade III in 40% and 42% of their participants, respectively.

The efficacy of PRP was shown with a 42.8% improvement in hair density after six months of treatment, according to a study by Butt et al. [12]. However, there is little literature on the efficacy of combination therapy with PRP and various topical agents. Our study compared the efficacy of combination therapy of topical formulations of PRP and Procapil with a topical formulation containing a mixture of redensyl, saw palmetto, and biotin.

The mean percentage of AGA improvement at the end of the second follow-up visit (after six months) was 11.9% in patients treated with PRP and Procapil (topical) and 21.9% in patients treated with PRP and RSB (topical). This difference was statistically significant (P-value < 0.00001). The study where Evron et al. [8] evaluated the efficacy of topical saw palmetto monotherapy for AGA showed an average AGA improvement of 27% at the end of 50 weeks. From the above findings, the AGA improvement, which was attained at 50 weeks using topical saw palmetto monotherapy, was attained in half the time (24 weeks) due to the adjuvants used along with topical saw palmetto, similar to the PRP procedure with redensyl and biotin. The efficacy of topical Procapil monotherapy or combination therapy with PRP is not documented. However, the study conducted by Karaca et al. [3], where a combination of topical redensyl, capixyl, and Procapil was used for AGA, showed an efficacy of 64.7%, which was assessed using a researcher evaluation score at the end of 24 weeks. Thus, the combination of Procapil and RSB (our two groups) appears to offer synergistically enhanced AGA therapy.

In our study, patients treated with the PRP and topical RSB combination showed higher satisfaction than those treated with the PRP and Procapil combination, as assessed using the visual analog scale (VAS). This finding is consistent with the findings of Evron et al. [8] who recorded greater patient satisfaction in AGA patients treated with saw palmetto.

The high AGA improvement rate with PRP and topical RSB combination therapy is due to the antiandrogenic properties of saw palmetto supplemented with redensyl, biotin, and PRP, which showed increased hair stem cell activity. Although topical Procapil has antiandrogenic properties, topical Procapil and PRP were not as effective on patients as the PRP and topical RSB, although they showed significant improvement from baseline.

Study limitations

The small sample size and the lack of trichoscopy/histopathology in assessing hair growth pre- and posttreatment were the main limitations of our study.

## Conclusions

The results of our study indicate that PRP in combination with topical redensyl, saw palmetto, and biotin is a more effective treatment than PRP in combination with topical Procapil. PRP is a widely employed treatment modality for hair restoration. There is a paucity of literature regarding the combination therapy of PRP with various topical agents. The combination of the above therapies not only has the capability to boost hair growth by regulating the hair cycle but also inhibits progressive hair loss.

Therefore, a combination therapy of PRP and topical RSB may be an innovative and effective approach for AGA.

This is the first conducted study that sheds light on the combination of PRP with various commercially available topical agents and provides a comparison of their efficacy.
